# Association between Fur Animal Necrotizing Pyoderma in breeding farm mink (*Neovison vison*) and reduced fertility

**DOI:** 10.1186/s13028-020-00564-w

**Published:** 2020-12-03

**Authors:** Oliver Legarth Honoré, Ida Sebbelov, Agnethe Wallin, Annemette Petersen, Tove Clausen, Peter Foged Larsen, Anne Sofie Hammer

**Affiliations:** 1 Department of Veterinary and Animal Sciences, Faculty of Health and Medical Sciences, University of Copenhagen, Ridebanevej 3, 1870 Frederiksberg C, Denmark; 2 Kopenhagen Forsking, Agro Food Park 15, 8200 Aarhus N, Denmark

**Keywords:** *Arcanobacterium phocae*, Cohort-study, Co-infection, FNP, Infectious diseases, *Streptococcus halichoeri*

## Abstract

**Background:**

The disease Fur Animal Necrotizing Pyoderma (FNP) has since 2000 been reported in many fur producing countries including Canada, Finland and Denmark. Development of FNP is characterised by rapidly forming treatment-resistant wounds on paws and in the head region. Economic losses related to FNP have been associated with mortality and decreased fur quality as well as increased veterinary costs. Also it has been suggested that FNP may be associated with reduced production results for breeding mink. The aim of this study was to evaluate if there is an association between FNP lesions in breeding animals and reduced production results based on a retrospective cohort study.

**Results:**

1465 breeding animals (244 males and 1221 females) were followed during the breeding season 2019 on five Danish mink farms. Two farms were removed from the analysis since no occurrence of FNP appeared in the observation group. After exclusion, 846 breeding animals (148 males and 698 females) remained in the analysis and were divided into two groups: exposed (EXP) or non-exposed (N-EXP) depending on the disease history of the males during mating. Females exposed to FNP positive males during breeding in average produce 14% fewer kits (P = 0.032) and these females were also more than double as likely to produce small litters (N ≥ 3) than N-EXP females. Female’s from the EXP group were introduced more times to males than females in the N-EXP group (P = 0.0001, 2.5 more times in average). Females in the EXP group did not have a statistically higher risk of becoming barren (P = 0.138) though the relative risk of becoming barren was 77% higher after encountering a FNP male.

**Conclusions:**

This study shows that FNP has more economic losses for the farms than direct loss of animals. Females in contact with males with FNP lesion during breeding have a higher risk of becoming barren, and produce significantly fewer kits compared to females whom haven’t been in contact with a FNP positive male.

## Background

Fur Animal Necrotizing Pyoderma (FNP) is an emerging bacterial disease of fur animals reported from several fur producing countries including Canada, Finland and Denmark [1–3]. We prefer using the term Fur Animal Necrotizing Pyoderma (FNP) instead of the previously applied Fur Animal Epidemic Necrotic Pyoderma (FENP), primarily because the disease tends to present with an endemic or sporadic pattern, rather than an epidemic pattern on most Danish mink farms. Development of FNP is characterised by rapidly forming treatment-resistant wounds on the paws and in the head region [2, 4]. FNP is associated with bacterial infections caused by *Arcanobacterium phocae* in synergistic effect with a *Streptococcus* spp. [2–4]. *A. phocae* has also been detected by polymerase chain reaction analyses in healthy minks suggesting that it is an opportunistic pathogen [2]. Through the last decade, FNP has become a disease of great concern for mink farmers worldwide, from both a welfare and economic perspective. Economic costs related to FNP have been associated with mortality and decreased fur quality as well as increased veterinary costs [2, 3]. It has also been suggested that FNP may be associated with increased numbers of barren females and reduced numbers of kits for breeding mink [2].

Spreading of FNP has generally been attributed to movement of animals, but transmission routes and port of entry remain unknown [3–5]. After weaning, mink are placed in cages with 2–4 animals and these mink will not be in direct contact with other mink during the growth season. During the mating season, females are moved between cages and thereby encounter several males. Farms with a higher number of breeding animals have been shown to exhibit a higher risk of FNP [6]. Some studies suggest a seasonal pattern where most cases of FNP were present in the winter or breeding season [1, 2, 4].

As no effective prevention or treatment is available, the current recommendation is to euthanize animals with lesions characteristic for FNP (wounds in the head and paw regions), though it is not unusual that breeding males and females with smaller FNP type lesions are allowed to breed (personal communication with breeders). On farms with high prevalence of FNP, culling of breeding animals can be a considerable economical issue, diminishing both breeding stock and the potential offspring from these. The aim of the study was to evaluate a possible association between FNP lesions in breeding animals and reduced production results in a retrospective cohort study. The males and females were followed throughout the breeding season, any presence of FNP-type lesions and the number of barren females and kits per female as well as encounters between animals were recorded.

## Methods

### Study design and main objectives

This study was designed as a retrospective cohort study aiming to elucidate the effect of FNP on the reproduction of Danish farm mink (*Neovison vison*). Based on recording of breeding contacts made during the breeding season (March 2019), the females were divided into 2 groups: exposed (EXP) and non-exposed (N-EXP). Females were characterized as EXP if they at any time during mating were placed in a cage with a male, who at any time point during the study period was diagnosed with FNP (FNP positive males). Females without any encounters with FNP positive males were characterized as N-EXP. FNP was diagnosed by the characteristic patoanatomical presentation (evaluated by trained veterinarian students) and using traditional microbiological methods as described below.

Beginning in March 2019, this study included 1465 animals on five farms (referred to as A–E). These farms were selected because they had a farm diagnosis of FNP and a high prevalence of the disease in autumn 2018. The five farms were distributed in Jutland and connected to three different feed kitchens. The farms were all medium sized (farm size measured in approximate number of breeding females, A: 4000, B: 1900, C: 3000, D: 3300, E: 4000). All farms used the same breeding system, referred to as “female rotation system” in this study, where five to six males were located in cages with five or six times as many females (25–36) in the cages around them. The females were transferred to a cage with a male, where they were giving the chance to mate. This transfer of females were counted as one encounter in this study. Not all encounters led to mating, resulting in different numbers of encounters between animals. After the encounter, successful or not, the females were moved out of the male’s cage and a new female was introduced. All encounters were recorded, as well as their order. The mink were followed from start of mating (1st of March) until all mink kits were born (10th of May). All farms were instructed to conduct mating as previous years. Moreover, they removed mink with wound prior to and during mating as soon as they were observed. The 1465 animals (244 males and 1221 females) were of five different colour types (Brown, Silver, Sliverblue, Palomino and Pearl beige). The beige colour types, Palomino and Pearl beige, are mixed during mating and are therefore included as one group in this study. The mink studied on each farm were all of the same colour type (the colour type with the history of highest prevalence of FNP type lesions in the previous year). On each farm, between 46 and 51 males were included in the study. During the study period, male and female mink on the farms were observed at least twice a day during mating and once daily after mating. All signs of disease among the mink were recorded during the observation period. The observation period was until euthanization for the males (after mating) and until birth of litters for the females. Swabs for microbiological culture were collected from all wounds. Wounds were identified as FNP lesions based the occurrence of necrotizing and exudative wounds, location on the paws or in the head region, and microbiological culturing of *A. phocae* and/or *Streptococcus halichoeri*.

### Microbiology

The collected swabs were spread on blood agar consisting of blood agar base (Oxoid Ltd, Basingstoke, UK) supplied with 5% sterile bovine blood. These were incubated for 48 h at 37 °C under anaerobic conditions using Anaerogen TM Atmosphere Generation System (Oxoid Ltd) according to the directions of the producer. Anaerobic incubation was used in order to reduce swarming of *Proteus*. Matrix-assisted laser desorption/ionization time of flight (MALDI-TOF Vitek MS system) was used for identification of bacterial isolates as previously described [7].

### Data collection

During the observation period, all signs of disease were recorded and evaluated. After mating season, females were divided into the two groups EXP and N-EXP, depending on the observation of FNP in the males. In May, after birth of kits, the number of barren females, kits per female, as well as the exchange of kits between females and kit mortality was recorded for each female. Barren females were in this study defined as females with no kits at first counting. Since it is not common practice to test if a female mink is pregnant and because female mink often eat there kits if they die in the perinatal period, it is not possible to differ between females losing a litter and females with an unsuccessful mating.

### Exclusion criteria

Two farms (D and E) were excluded from the study since no FNP cases were recorded in the selected study group during the observation period (N = 481 females). Females from the remaining three farms (A, B and C) with other illnesses, types of wounds or trauma as well as females succumbed to peripartum mortality (N = 42) were excluded from the data analysis.

### Statistical analysis

The data were analysed as retrospective cohort data, where the primary outcome was the number of kits born. In this study the statistical significant cut-off P-value was ≤ 0.05. Data analysis was performed using R Statistics [8].

Numbers of kits were investigated using a negative binomial model with number of kits born as response variable and assigned group and number of encounters as explanatory variables. The variable describing number of encounters was transformed into a factor variable, separating by the 3^rd^ quantile. This new variable then described if the number of encounters was normal or high. A binomial logistic regression model was used to assess if females in the EXP group produced less kits compared to females in the N-EXP group. Statistics were used to calculate the relative risk (RR) for females to become barren or producing small litter sizes (N ≤ 3) when exposed to a FNP affected male.

### Ethical statement

All national guidelines and laws for the care and use of animals were followed during the study. The animals were inspected daily and animals with wounds received treatment according to national regulation and the Code of Practice of the Danish Fur Animal breeders.

## Results

A total of 148 male and 698 female mink from three farms (A–C) were included in the analyses. The distribution of mink in farms and groups are presented in Table 1. Eight males and six females were diagnosed with FNP. The distribution of the eight males were four on farm A, one on farm B and three on farm C. These eight males encountered 111 different females. Five of these females were excluded as a result of the exclusion criteria. In total, 106 females were assigned to the EXP group. The six females representing 1.16% of the total females were diagnosed with FNP after the mating season and remained in the study. The distribution of the six females was 2 females in the EXP group on farm A and 4 females in the N-EXP group on farm C. All six females were barren.

**Table 1:** Production results from the three farms

Farm	A		B		C		Total	
Colour type	Beige		Silverblue		Brown			
Group	N-EXP	EXP	N-EXP	EXP	N-EXP	EXP	N-EXP	EXP
No. BF	116	47	213	12	181	21	510	80
No. Kits	746	277	1585	79	1403	162	3734	518
No. BaF	26	15	34	3	22	8	82	26
Avg. kits per BF	6.43 5.99;6.87^C^	5.89 5.16;6.63^C^	7.44 7.09;7.79^C^	6.58 5.24;7.92^C^	7.75 7.44;8.07 ^C^	7.71 6.42;9.01^C^	7.32 7.11;7.53^C^	6.47 5.89;7.06^C^
Avg.E to male	5.60 5.13;6.12 ^C^	8.10 7.00;9.22^C^	3.36 3.13;3.58 ^C^	4.47 3.47;5.47^C^	4.40 4.15;4.61^C^	5.20 4.60;5.81^C^	4.3 4.07;4.44 ^C^	6.8 6.06;7.54^C^
Avg.E to FNPm	0	1.5 1.26;1.68^C^	0	1.5 1.18;1.89^C^	0	1.2 1.03;1.32^C^	0	1.4 1.26;1.53^C^
Pct. BaF	18.3%	24.2%	13.8%	20.0%	10.8%	27.6%	13.8%	24.5%

It shows an overview of the production results and average kits per breeding female from the three farms included in this field study. The data were divided into the different farms as well as into the two groups exposed (EXP) and non-exposed (N-EXP)

No., numbers of.; BF, Breeding females; BaF, Barren females; Avg.E, Average encounter; FNPm, males with lesions diagnosed as FNP; Pct. , percentage; ^C^, 95% confidence interval

The production results for the study groups are summarized in Table 1. The females in the EXP group were introduced to males 2.5 more times in average (6.8 encounters) compared to the N-EXP group (4.3 encounters). This difference is significant (P = 0.00001). In average, the females in the EXP group encountered FNP males 1.4 times. The age of the females was significantly different between the two groups (P = 0.006). In order to calculate the significance of the other differences, the number of encounters as well as the age difference have been taken into account in all other statistical calculations.

Even though females in the EXP group apparently had a higher percentage of barren females compared to females in the N-EXP group (24.5% vs 13.8%), there was no statistical significance (P = 0.138) when calculating on the entire dataset using logistic regression. On the individual farms, P = 0.819, P = 0.900 and P = 0.021 for farm A, B and C respectively, only farm C has a significant higher percentage of barren females in the EXP group compared to the N-EXP group. Even though there was not significantly more barren females in the EXP group, the risk of a female becoming barren was higher in the EXP group, with a relative risk of 1.77.

Female mink in the EXP group produced significantly fewer kits (14% fewer kits in average, P = 0.032). Using a negative binomial model it was calculated that mated females in the EXP group on average gave birth to 14% fewer kits compared to the N-EXP group. When removing barren females from the equation, the dataset was too small to perform a negative binomial model. In order to investigate if breeding females in the EXP group produced significant fewer kits, Chi-squared distribution and Mann–Whitney U test were performed. Both of these methods showed a significantly lower reproduction result in females in the EXP group compared to the N-EXP group, P = 0.027 and P = 0.011 for Chi-squared and Mann–Whitney U test respectively. Furthermore, the risk of females producing a small litter size (N ≤ 3), was higher in the EXP group with a relative risk of 2.11.

When comparing the three farms average kits per mated female and number of barren females to the national production results (collected by Kopenhagen Fur on the basis of 2.1 million female mink), we found that the study farms were overall performing close to the national average, see Table 2. This is the case, both when comparing the production results within colour types or regardless of colour type (column named “Total” in Table 2).

**Table 2:** Results from Farm A–C compared to national average

Colour type	Pearl beige/palomino		Silverblue		Brown		Total	
	Avg. Kits	Pct. BaF	Avg. Kits	Pct. BaF	Avg. Kits	Pct. BaF	Avg. Kits	Pct. BaF (%)
National average/mated female	5.12/5.33	10.12%/8.52%	5.66	7.64%	5.6	7.64%	5.33	8.69
Farm A	5.12/5.61	10.65%/6.67%	–	–	–	–	5.33	8.90
Farm B	–	–	5.7	7.7%	–	–	5.01	9.90
Farm C	–	–	–	–	6.1	4.9%	6.01	10.5

It shows the production result from the different farm and compares them to the national average. *Avg. Kits is the average kits per mated female. BaF = percentage of barren females. The national average is data from Kopenhagen fur based on 2.1 million mink. For the individual farms A–C (the last three rows), the data on the different colour types are based upon all mink of same colour, not just the ones included in this study, e.g. all brown mink on farm C. The data in the “Total” column are the combined results of all colour types on the farm

## Discussion

On three of the five farms included in the data set, breeding animals with FNP were recorded. On these three farms, the 148 male breeders and 698 female breeders included in this study produced a total of 4252 mink kits. Among these breeding animals, 5.41% of the males and 1.16% of the females developed FNP. Two of the farms were not included in the data analysis because no FNP lesions were observed on the selected groups during the observation period. It is worth noticing, that not all farms with a farm diagnosis of FNP will experience FNP in the breeding animals. The mechanisms behind variation in the occurrence of FNP between individuals and farms remain unknown. Suggested factors include genetics, immunological robustness and transfer of mink [6, 9].

Females in the EXP group had significantly more encounters with males than the females in the N-EXP group (P = 0.00001). 1.4 of the 2.5 more encounters in average can properly be explained by the direct encounter to FNP positive males. FNP positive males are affected physically with pain and soreness and perhaps fever which can result in unsuccessful mating and thus result in more movement of the females. The remaining difference in encounters can be an effect of many different variables; males with general bad sperm quality, females whom are bad at mating etc. These variables are not possible to explain with the data from this study. However a female whom encounters more males will have a higher risk of encounter a FNP male.

There were no overall significant differences between the two groups with respect to the quantity of barren females (P = 0.138). Farm C was the only farm with a significant difference (P = 0.021). It is however not possible to determine if this significance was an effect of differences in farm management or a result of the limited numbers of barren females in each group, increasing the risk of a false positive result. Even though the overall difference was not significant, the risk of becoming barren for females encountering a FNP male were calculated to a relative risk of 1.77. This indicates that females in the EXP group have 77% higher risk of being barren, compared to the N-EXP group. Interestingly the six females, which developed FNP were all barren. Therefore, it could be speculated that FNP in males as well as females can be associated with poor production results. This would be relevant to further investigate.

Females in the EXP group had significantly reduced reproduction results (kits per mated females) compared to females in N-EXP groups (P = 0.032). The average difference in litter sizes was calculated to be 14% fewer in the EXP group. When removing the barren females from the equation and thus calculate the reproduction results as kit per breeding females, the dataset was too small to make a negative binomial model and thus Chi-squared and Mann–Whitney U tests were performed instead. The negative binomial model is a stronger statistical test and are preferred when possible. Both Chi-squared and Mann–Whitney U test resulted in statistical significant results with a P-value of 0.027 and 0.011 for respectively. The relative risk for females to produce small litters (N ≤ 3) were calculated to be 2.11 in this study. This indicates that females in the EXP group are more than double as likely to produce small litters, compared to females from the N-EXP group.

The results show that FNP has an effect on the breeding results and is consistent with previous observations by researchers and farmers that indicates that FNP may affect production results in breeding mink [2].

When looking at the average kits born per breeding female, the Silverblue mink seem to be more affected by exposure to FNP positive males than Palomino/Pearl beige and brown mink. In average Silverblue mink produced 0.86 less kits per breeding females in the EXP group compared to the N-EXP group. For the Palomino/Pearl beige and Brown mink this number were 0.54 and 0.04 respectively. This could indicate that Brown mink may be more resistant towards FNP compared to the other colour types; an observation also presented by Nordgren et al. [6]. The above mentioned difference may have been affected by the fact that this study was conducted on different farms with potentially different strategies, methods and different feed kitchens. In order to evaluate differences in colour types the optimal study design would include several colour types on the same farm. To minimize farm related differences and in order for the results to be representative for Danish mink production, the included farms were average size farms with standardized production systems, including cages, food and watering systems and they were applying routine breeding methods. Furthermore, Table 2 shows that all three farms are close to the national average in respect to average kits per mated females both on the selected colour types and on the whole farm regardless of colour.

During the very short breeding season of mink, reproductive efficiency is of great importance if key reproduction targets are to be achieved. Further studies including more farms and several colour types housed on the same farms, are necessary in order to further investigate the effect of FNP on mink breeders and the mechanisms behind the apparent negative effect on breeding results. It may be speculated that infection with bacterial agents may cause fever in the male mink and thereby affect semen quality, however this needs to be further studied. Case studies in humans have demonstrated that fever can have latent effects on sperm chromatin structure and may result in transient release of abnormal sperm there by affecting fertility [10, 11].

## Conclusion

Females in the EXP group produced significantly fewer offspring than females in the N-EXP group. The difference was calculated to be 14% fewer kits per mated female in average. When barren females were removed from the equation, females in the EXP group still produced significantly fewer kits. This result is supported by the higher relative risk of 2.11 for females to produced small litters.

Females in the EXP group encountered significant more males, in average 2.5 more encounters.

Even though there was not a significant difference in the numbers of barren females between the two groups, the risk of a female becoming barren was higher in the EXP group with a relative risk of 1.77.

These results indicate that FNP may be associated with considerable economical loss due to reduced production results. To our knowledge, this is the first study to confirm a supposed effect of FNP on the production results of breeding farm mink.

## Data Availability

Data are available from the corresponding author upon request.

## Abbreviations

Avg: Average
FNP: Fur Animal Necrotizing Pyoderma
N: Numbers
RR: Relative risk
EXP group: Exposed group
N-EXP group: Non exposed group
